# The Role of Intraoperative Indocyanine Green (ICG) and Preoperative 3-Dimensional (3D) Reconstruction in Laparoscopic Adrenalectomy: A Propensity Score-matched Analysis

**DOI:** 10.1097/SLE.0000000000001105

**Published:** 2022-10-03

**Authors:** Giuseppe Palomba, Vincenza Paola Dinuzzi, Francesca Pegoraro, Roberto Ivan Troisi, Roberto Montalti, Giovanni Domenico De Palma, Giovanni Aprea

**Affiliations:** † Division of Endoscopic Surgery; † General and Oncology Surgery Department, Ospedale Giuseppe Fornaroli, Magenta, Mi; ‡ Division of Hepato-Biliary-Pancreatic, Minimally Invasive and Robotic Surgery and Kidney Transplantation; § Department of Public Health, Federico II University, Naples Italy

**Keywords:** indocyanine green fluorescence, preoperative planning, three-dimensional reconstruction, laparoscopic adrenalectomy, general surgery, propensity score matching analysis

## Abstract

Laparoscopic adrenalectomy (LA) is considered the “gold standard” treatment of adrenal lesions that are often coincidentally diagnosed during the radiologic workup of other diseases. This study aims to evaluate the intraoperative role of indocyanine green (ICG) fluorescence associated with preoperative 3-dimensional reconstruction (3DR) in laparoscopic adrenalectomy in terms of perioperative outcomes. To our knowledge, this is the first prospective case-controlled report comparing these techniques.

**Materials and Methods:**

All consecutive patients aged≥18 and undergoing laparoscopic transperitoneal adrenalectomy for all adrenal masses from January 1, 2019 to January 31, 2022 were prospectively enrolled. Patients undertaking standard LA and those undergoing preoperative 3D reconstruction and intraoperative ICG fluorescence were matched through a one-on-one propensity score matching analysis (PSM) for age, gender, BMI, CCI score, ASA score, lesion histology, tumor side, and lesion diameter. Differences in operative time, blood loss, intraoperative and postoperative complications, conversion rate, and length of stay were analyzed.

**Results:**

After propensity score matching analysis, we obtained a cohort of 36 patients divided into 2 groups of 18 patients each. The operative time and intraoperative blood loss were shorter in patients of the 3DR group (*P*=0,004 and *P*=0,004, respectively). There was no difference in terms of length of stay, conversion rate, and intraoperative and postoperative complications between the 2 groups.

**Conclusions:**

The use of intraoperative ICG in LA and preoperative planning with 3DR images is a safe and useful addition to surgery. Furthermore, we observed a reduction in terms of operating time and intraoperative blood loss.

Adrenal incidentalomas are adrenal masses coincidentally diagnosed during the radiologic workup of other diseases. In most cases, adrenal incidentalomas are nonfunctioning adrenal adenomas, but they can also be adrenal tumors, pheochromocytomas, adenomas, or hormone-releasing metastases.^1^ Hyperfunctioning masses develop in ~1.7% of cases, with an increased risk in patients with lesions greater than 3 cm.^2^ Surgery is not usually indicated in patients with an asymptomatic unilateral adrenal mass <4 cm, nonfunctioning lesions, and evident benign features in imaging studies.^3^–^5^ Adrenalectomy is indicated for pheochromocytomas, other symptomatic hormone-secreting tumors, those at high risk of malignancy, and large adrenal masses.^3^–^5^ Biochemical screening for tumor hypersecretion is mandatory in all adrenal incidentalomas.^3^,^4^ The diagnosis of pheochromocytoma is of primary importance due to its life-threatening complications. Tumor size and imaging characteristics of nonfunctioning adrenal incidentalomas must be carefully assessed because of their risk of transformation and subclinical hormone secretion.^2^,^6^,^7^ According to the ESE (European Society of Endocrinology) and ESNAT (European Network for the Study of Adrenal Tumors) guidelines of 2016 for incidentaloma management, a multidisciplinary tumor board is essential and requires the appraisal of experienced endocrinologists, radiologists, anesthesiologists, and surgeons.^3^,^8^

Laparoscopic adrenalectomy is considered the “gold standard” treatment of adrenal lesions.^9^ In the last few years, indocyanine green (ICG) has been gaining more and more attention as an innovative instrument for this type of surgical procedure.^10^–^15^ The use of ICG in adrenal surgery is beneficial for 2 main reasons: first, it provides a contrast distinction between highly vascular and hyperfluorescent adrenal tissue and less vascular, hypofluorescent retroperitoneal tissue, which helps during dissection.^10^,^15^ Secondly, it can guide partial adrenalectomy easing the identification of healthy adrenal tissue and tumor, especially during resections of pheochromocytomas.^10^,^11^,^15^ However, this technique has been widely developed in ophthalmology, neurosurgery, thoracic surgery, colorectal surgery, and hepatobiliary surgery, while for adrenalectomy, there are few studies only.^10^,^16^–^21^

Three-dimensional reconstruction (3DR) is another useful tool that could improve laparoscopic adrenalectomy.^22^ This technology generates high-quality 3D images from radiologic 2D images obtained during radiologic workup through computed tomography (CT) or magnetic resonance (MR).

Some surgical series support the association of ICG and 3D reconstruction, and their benefits appear to particularly combine during complex hepato-biliary procedures.^23^–^25^

This study aims to evaluate the intraoperative role of ICG fluorescence and preoperative 3D reconstruction in laparoscopic adrenalectomy in terms of perioperative outcomes.

## MATERIAL AND METHODS

### Study Population

**FIGURE 1:**
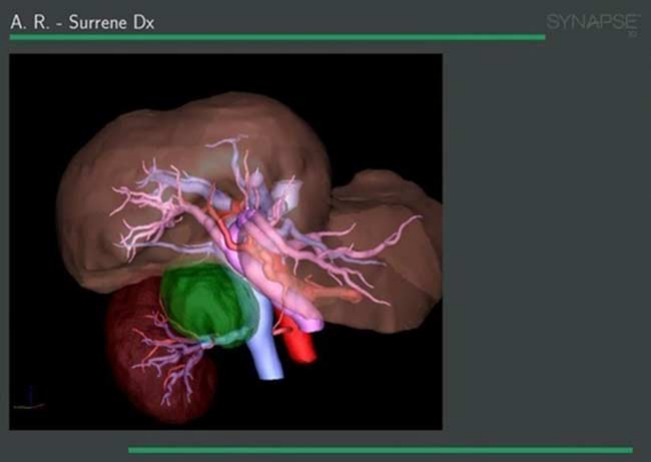
3D reconstruction of a right adrenal pheochromocytoma, as shown in the exported PDF file utilized during the surgical procedure. Each structure is shown in a different color and can be shown, sheered, or hidden depending on the necessity. The whole reconstruction can be zoomed in and rotated in all directions.

**FIGURE 2:**
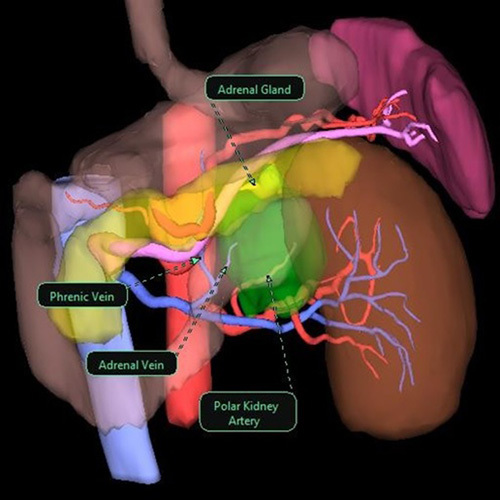
3D reconstruction of a left adrenal pheochromocytoma, as visualized in the Software before PDF exportation. Every structure identified can be pointed out with a label.

All consecutive 62 patients aged≥ 18 and undergoing laparoscopic transperitoneal adrenalectomy (LA) for all adrenal masses at the General Surgery Unit of the University Hospital Federico II of Naples from January 1, 2019 to January 31, 2022 were prospectively enrolled in this study. Patients with a history of abdominal surgery and severely obese patients (Body Mass Index >40 Kg/m^2^) were excluded. The following perioperative outcomes were analyzed: conversion rate, intraoperative blood loss, operative time, length of stay, size of the specimen, and intraoperative or postoperative complications (based on Clavien-Dindo classification).^26^ Blood loss was measured by evaluating the blood contained in the aspirator reservoir, excluding the irrigation fluids used for peritoneal washing. In case of very low blood loss, estimation of the gauzes used for hemostasis was performed. This prospective study was performed according to the Declaration of Helsinki principles, and approval was obtained from the Institutional Review Board and Ethics Committee and each patient through specific, informed consent. Patients were divided into 2 cohorts: the 3DR group benefited from a preoperative 3D reconstruction and an intraoperative ICG injection, while the remaining patients underwent standard laparoscopic treatment. The cases performed with ICG-3D were randomly enrolled at the same time as the control cases (ie, without ICG-3D). During preoperative planning, each patient in 3DR group underwent an injected triphasic CT scan. The images were loaded in Digital Imaging and Communications in Medicine (DICOM) file extension on a dedicated PC (Dell Precision 7820 – Intel Xeon Silver 4114 CPU 2.20 GHz and 2.19 GHz Double Processor – 32 GB RAM – Windows 10 Pro) and elaborated with a 3D Software (Synapse 3D, Fujifilm, Tokyo, Japan). A surgeon or a resident surgeon under the supervision of a staff surgeon was responsible for the reconstructions. Before each surgical procedure, a PDF file of the reconstruction was exported and shown on a dedicated screen in the operating room, allowing intraoperative manipulation of the model (as shown in Fig. 1 and Fig. 2).

**FIGURE 3:**
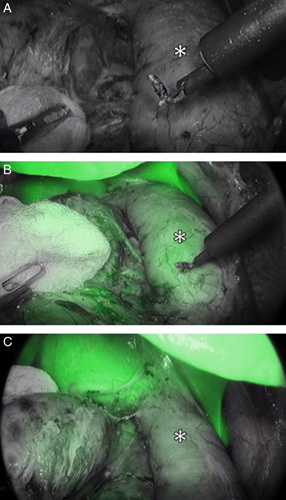
Acquisition at time zero of ICG administration during adrenalectomy for right pheochromocytoma (**A**). Progression of the dye at 30” (B) and 60” (**C**) from the administration with visualization of the adrenal vein, the vena cava (white asterisk), the renal vein, and the hypofluorescent lesion.

**FIGURE 4:**
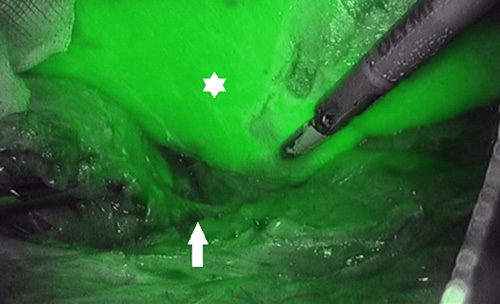
Acquisition at 60” of a Cushing lesion: the adrenal vein is clearly visible (white arrow). The liver (white asterisk) is highly fluorescent due to ICG’s metabolism.

Each 3DR group patient was administered an intradermal test dose of 0.1 mg of ICG the day before surgery to rule out any allergic reactions. Intraoperatively, after adhesions ablation and at an early stage of the dissection phase, an initial intravenous dose of 5 mg of ICG diluted in 10 cc of distilled water was administered in 5 seconds to enhance the gland and its vascularization. If the resulting fluorescence was not sufficiently contributive, a second ICG bolus was administered. A laparoscopic ICG-filtered optic system (Stryker Precision Ideal Eyes HD Laparoscope, 5.5 mm, 30 mm length, 30 degrees) was used to visualize the resulting fluorescence, as seen in Figure 1 and Figure 2. In patients with large right adrenal gland tumors without anatomic plane alteration, dissection continued according to the standard laparoscopic adrenalectomy technique following the main landmarks.^27^–^29^ The vascular enhancement onset is 10 to 20 seconds after the injection and lasts between 30 and 75 seconds after each ICG bolus. The administered ICG dose and the surgical stage at the time of injection were registered, and the relative images were saved (Fig. 3A-C and Figure 4). We observed that medullary tumors such as pheochromocytomas were hypofluorescent at ICG, while cortical tumors such as Cushing were hyperfluorescent.

### Statistical Analysis

Categorical data were reported as frequencies and percentages and comparisons between groups were performed using the χ^2^ test with Yates’ correction or Fisher exact test when appropriate. Continuous variables were reported as mean±SD and were compared using the 2-sided Student t test for normal distributions; nonparametric variables were reported as median and ranges and were compared using the Wilcoxon-Mann-Whitney *U* Test.

The 2 study groups were compared by minimizing the different distribution of covariates through a propensity score matching analysis (PSM) with a caliper width of 0.20, obtaining a one-to-one match, and excluding patients in whom the PSM was not applicable. The model was based on logistic regression by using the single nearest neighbor matching method without replacement (no repetition of a patient in either group), until all possible matches had been formed. The 2 groups were matched for age, gender, BMI, CCI score, ASA score, lesion histology, tumor side, and lesion diameter. Statistical significance was set at *P*<0.05.

Statistical analysis was performed using the IBM SPSS Statistics for Windows, version 20.0. Armonk, NY, IBM Corp. IBM SPSS Statistics version 26 (SPSS Inc. Chicago, IL).

## RESULTS

We performed 62 LA from January 1, 2019 to January 31, 2022: all the procedures were performed by an experienced surgeon (A.G.). Twenty-five patients were treated using IGC and 3D reconstruction and 37 underwent standard treatment. Patients’ characteristics are summarized in Table 1. We performed 3 bilateral laparoscopic adrenalectomies (2 Cushing, and 1 pituitary adenoma). Intraoperative complications included: perforation of a 10 cm kidney cyst, nephrectomy, caval lesion, and cardiac arrest during an 8 cm pheochromocytoma resection, which resulted in the patient’s death. The latter patient had 3 heart attacks in the past and suffered from treatment-unresponsive high-grade hypertension. Postoperative complications were mainly treated conservatively and included mild fever, hypertensive crisis, postoperative anemia, vomiting, and nausea. One pregnant patient with right pheochromocytoma (treated through standard treatment) suffered from immediate postoperative anemia and received a blood transfusion; she was discharged on POD3 without further complications. Severe postoperative complications included a case of kidney failure for thrombosis of the right renal vein, treated with dialysis seances.

**TABLE 1:** Characteristics of all Patients Undergoing Laparoscopic Adrenalectomy

Total LA Patients (n=62)	
Males (%)	20 (32.3)
Age (y)	49.7±12.7
BMI (Kg/m^2^)	26.2 (22.0–34.6)
CCI Score	2 (0–9)
ASA Score	
II	31 (50.0%)
III	26 (41.9%)
IV	5 (8.1%)
Lesion Histology	
Cushing Syndrome	21 (33.9%)
Non-Secreting Adenoma	21 (33.9%)
Pheochromocytoma	16 (25.8%)
Other	4 (6.4%)
Side	
Left	28 (45.1%)
Right	31 (50.0%)
Bilateral	3 (4.8%)
Lesion diameter (cm)	5.9 (2.0–13.0)
Surgical time (min)	66 (35–180)
Blood loss (ml)	10 (0–750)
Intraoperative complications	4 (6.4%)
Transfusions	2 (3.2%)
Conversion	3 (4.8%)
Postoperative complications	13 (20.9%)
CD<3	12 (19.3%)
CD≥3	2 (3.2%)
Length of stay (days)	4 (2–7)
Death	1 (1.6%)
Intraoperative	1 (1.6%)

ASA indicates American Society of Anesthesiologists; BMI, Body Mass Index; CCI, Charlson Comorbidity Index; CD, Clavien-Dindo Score; LA, Laparoscopic Adrenalectomy.

The 2 cohorts of patients significantly differed in CCI scores and exhibited a slight disparity in sex and age (Table 2). After applying one-on-one PSM, we obtained a total of 36 patients divided into 2 groups of 18 patients each, which showed no differences in demographic, preoperative, and adrenal mass characteristics. Patients in the 3DR group included 11 left, 6 right, and 1 bilateral LA, while patients treated through a standard treatment comprised 10 left, 7 right, and 1 bilateral LA (Table 2).

**TABLE 2:** Characteristics of all Patients Undergoing Laparoscopic Adrenalectomy Before and After the Application of a Propensity Score Matching Analysis

	Patients Characteristics Before PSM (n=62)			Patients Characteristics After PSM (n=36)		
	3DR Patients (n=25)	Standard Treatment (n=37)	*P*	3DR Patients (n=18)	Standard Treatment (n=18)	*P*
M (%)	11 (44.0%)	9 (24.3%)	0.165	8 (44.4%)	7 (38.9%)	>0.999
Age (y)	51.8±12.1	48.3±13.2	0.365	50.4±13.2	48.6±12.1	0.689
BMI (Kg/m^2^)	26.0 (22.0–34.6)	26.1 (22.0–33.0)	0.762	24.6 (22.0–31.0)	25.8 (22.0–33.0)	0.491
CCI Score	3.0 (0–9)	1.0 (0–7)	**0.024**	2 (0–6)	2 (0–7)	0.724
ASA Score, n (%)						
II	11 (44.0)	19 (51.4)		10 (55.6)	8 (44.4)	—
III	11 (44.0)	16 (43.2)	0.637	7 (38.9)	8 (44.4)	0.793
IV	3 (12.0)	2 (5.4)	—	1 (5.6)	2 (11.1)	—
Lesion histology, n (%)						
Cushing Syndrome	7 (28.0)	14 (37.8)	—	5 (27.8)	6 (33.3)	—
Non-secreting adenoma	8 (32.0)	13 (35.1)	0.715	6 (33.3)	6 (33.3)	>0.999
Pheochromocytoma	8 (32.0)	8 (21.6)	—	6 (33.3)	5 (27.8)	—
Other	2 (8.0)	2 (5.4)	—	1 (5.6)	1 (5.6)	—
Side, n (%)						
Left	12 (48.0)	16 (43.2)	—	11 (61.1)	10 (55.6)	—
Right	12 (48.0)	19 (51.4)	0.910	6 (33.3)	7 (38.9)	>0.999
Bilateral	1 (4.0)	2 (5.4)	—	1 (5.6)	1 (5.6)	—
Lesion diameter (cm)	5.5 (3.5–9.5)	6.0 (2.0–13.0)	0.799	5.2 (3.5–9.5)	5.7 (3.0–8.0)	0.809
Surgical time (min)	69 (35–160)	77 (40–190)	0.266	53.5 (35–140)	74 (40–180)	**0.040**
Blood loss (ml)	5 (0–250)	30 (0–750)	0.094	0 (0–250)	40 (0–750)	**0.040**
Intraoperative complications, n (%)	1 (4.0)	3 (8.1)	>0.999	1 (5.6)	1 (5.6)	>0.999
Conversion	1 (4.0)	2 (5.4)	>0.999	0	1 (5.6)	>0.999
Postoperative						
Complications, n (%)	4 (22.2)	5 (27.8)	—	4 (22.2)	5 (27.8)	—
CD<3	4 (22.2)	4 (22.2)	>0.999	4 (22.2)	4 (22.2)	>0.999
CD≥3	0	1 (5.6)	—	0	1 (5.6)	—
Length of stay (days)	3.6±0.9	4.1±1.9	0.275	3.6±0.9	4.0±1.7	0.372

Bold values are statistically significant.

3DR indicates patients undergoing preoperative 3D Reconstruction and Indocyanine Green intraoperative enhancement; ASA, American Society of Anesthesiologists; BMI, Body Mass Index; CCI, Charlson Comorbidity Index; CD, Clavien-Dindo classification; PSM, Propensity Score Matching Analysis.

The operative time was significantly shorter in patients of the 3DR group (*P*=0.04) (Table 2). Furthermore, our data suggest that 3DR contributes to reducing intraoperative blood loss (*P*=0.04) (Table 2). There was no difference in terms of length of stay, conversion rate, and intraoperative and postoperative complications between the 2 groups. We observed 1 conversion in the standard treatment group due to a caval lesion. In the 3DR group, 1 patient experienced the perforation of a kidney cyst without conversion. We observed 4 postoperative complications in the 3DR group and 5 in the standard treatment group without a statistically significant difference. Specifically, we observed 3 complications of grade I (fever, nausea, and vomiting), and one of grade II (hypertensive crisis) in the 3DR group, while in patients undergoing the standard treatment, we observed 2 complications of grade I (fever and nausea), 2 of grade II (hypertensive crisis and transfusion) and 1 of grade IVa (kidney failure, treated with dialysis).

## DISCUSSION

Adrenalectomy is a rare procedure, with only 5323 cases performed in the United States in 2006.^30^ Open transperitoneal and retroperitoneal adrenalectomy have been the standard treatment for adrenal lesions for most of the twentieth century. Since Gagner performed the first laparoscopic total adrenalectomy in 1992, this approach has become the gold standard treatment of adrenal masses.^3^,^31^,^32^ This minimally invasive technique resulted in better intraoperative visualization of anatomic structures and lesions, lower intraoperative blood loss, lower complication rates, reduced postoperative pain, and a shorter hospital stay than the classic open approach. Since adrenalectomies usually account for a small part of a laparoscopic surgeon’s practice, it has been suggested that LA should be performed in specialized centers only, by advanced laparoscopy experts and in a context of multidisciplinary management.^33^

Moreover, the laparoscopic technique hampers the surgeon’s ability to receive tactile feedback, which is traditionally used to discern tumor edges and identify vascular structures in open procedures. In addition, the retroperitoneal space is narrow and barely reachable by laparoscopy.^34^–^36^ Given the anatomic differences between the right and left sides, laterality is an additional challenge.

The Society of American Gastrointestinal and Endoscopic Surgeons (SAGES) states that a sufficient level of expertise is reached after 20 to 40 controlled cases.^37^,^38^ Reflecting on their experience of laparoscopic adrenalectomy in a teaching hospital, endocrine surgery training programs described the identification of the left adrenal vein as one of the most challenging steps to be taught.^29^,^39^

The use of intraoperative ICG fluorescence could help reduce the learning curve for trainee surgeons and help expose anatomic variations to the experienced surgeon.^13^ Fluorescence-guided surgery could greatly help during the identification and dissection of critical vascular structures.^40^ Careful and quick recognition of the adrenal vein is important during the resection of pheochromocytomas due to the hemodynamic stress observed during tumor manipulation.^40^ In most cases, blood loss is minimal, with a median of 160 ml observed in all types of adrenal lesions.^29^ However, because of the proximity to the inferior vena cava on the right and the renal vein on the left, a critical error can cause considerable bleeding. Excessive bleeding has been reported to be the most common factor associated with conversion at an open laparotomy.^29^,^41^ Furthermore, damage to these vascular structures can lead to massive bleeding with the need for blood transfusions, the development of disseminated intravascular coagulation, gas embolism, and death.^41^

We hypothesize that the ability to better understand the vascular anatomy of adrenal lesions with near-infrared spectrum ICG fluorescence imaging improves surgical evaluation and dissection, and reduces vascular lesions.^42^ ICG is an FDA-approved nontoxic dye that emits a fluorescent signal when excited by NIR light (600 to 900 nm).^43^ It has a high safety profile and has been used clinically for more than 50 years for heart and liver function tests. After intravenous injection, the dye binds to plasma proteins and circulates through the intravascular space until it is absorbed by the hepatocytes and excreted exclusively through the hepatobiliary system.^29^ The intensity of the fluorescence signal is proportional to tissue’s blood flow, increasing for well-vascularized structures such as the adrenal gland. Intraoperative evaluation of ICG fluorescence can be done quickly and increases the operative time by a few minutes only.

Dip et al^44^ first demonstrated the feasibility of laparoscopic adrenal visualization using ICG imaging in a large animal sample. Manny et al^45^ used this method during robotic partial transperitoneal adrenalectomy in 3 patients with solitary adrenal lesions. By administering 5 mg of intravenous IGC, the adrenal masses showed different fluorescence intensities (hypofluorescent, isofluorescent, or hyperfluorescent) compared with normal adrenal tissue.^45^ Lerchenberger et al^46^ used ICG imaging during three laparoscopic partial adrenalectomies for bilateral pheochromocytoma and bilateral Cushing’s syndrome. Another important application of fluorescence is the evaluation of sufficient perfusion of the remaining adrenal tissue after partial adrenalectomy.^13^ DeLong et al^13^ showed visual enhancement of the adrenal vessels with clear demarcation of the adrenal gland and adjacent tissues in 4 laparoscopic adrenalectomies using fluorescence. Colvin et al^14^ applied ICG visualization to robotic adrenalectomies, finding that this technique was superior or equivalent to standard robotic view in 72.1% of their cases. In 2018 Kahramangil et al^47^ characterized 100 robotic adrenalectomies and described several fluorescence patterns exhibited by different anatomic features and adrenal pathologic conditions, helping to define the best clinical indications for ICG use. For example, the retroperitoneum tissue luminescence was transitory, while liver and adrenal parenchyma showed a more persistent fluorescence that lasted throughout the whole procedure. Healthy adrenal cortical tissue was hyperfluorescent in 74% of patients, and a different pattern indicated distinct histological origin as well (ie, medullary vs. cortical).^47^ Arora et al^15^ reported on 55 laparoscopic adrenalectomies using ICG images, with similar benefits to the previous study.

Another potentially helpful tool is the creation of preoperative 3D reconstructions that could guide the surgical gesture. At present, many software applications for surgical use are available (Myrian^©^ by Intrasense, Ziostation^©^ by Ziosoft, Synapse^©^ Vincent by Fujifilm, Liver Iqqa^®^ by Edda Tecnologia, Pathfinder Liver ScoutTM).^48^ This technology allows to obtain a basic anatomic scenery of each patient’s particular surgical site, and it is performed based on CT or MR images. These high-quality 3D images give precious information on tumors, vascularization, the relationship between organs, and the possible proximity to vessels or structures to preserve. Moreover, it could help surgeons with conventional image interpretation and implement preoperative planning in case of challenging conditions. The 3D image visualization showed significant advantages over standard 2D section visualization. This system has been widely developed in hepatobiliary surgery,^16^ while in adrenal surgery, there are still few cases in the literature. The application of 3D reconstruction in LA could ease the distinction of healthy tissue from adrenal adenoma in partial adrenalectomies, the identification of correct resection margins, and the visualization of the gland and adenoma vascular supply.^10^,^11^,^15^

Mitterberger et al^33^ evaluated the efficacy of 3D CT during preoperative planning of partial LA in a series of 12 patients: the reconstructions allowed better identification of adrenal glands and vascular systems, and tumors were discerned from normal adrenal glands more precisely. Moreover, 3D images were easily accessible thanks to “rotation” and “zoom” functions, simplifying the evaluation of complex anatomic relationships.^33^ Giant adrenal lesions could benefit from this approach as well, as described in a case report.^22^

The 3D images allowed us to accurately plan the surgery preoperatively (Figs. 2 and 3), while intraoperative ICG findings were a useful guide to assessing adrenal vascularization and lesions. Moreover, the more accurate preoperative and intraoperative study could shorten young surgeons’ learning curve.

Usually, mass size plays an important role in operative time. In this study, the 2 groups did not differ in terms of lesions’ size, but we observed a reduction in the operating time and blood loss with a statistical difference (*P*=0.04 in both cases). In this setting, our approach helps to identify anatomic structures quickly and precisely, reducing blood loss and operative time. The combination of these 2 methods allowed us to recognize intraoperatively the vascular structures (anatomic and variants) and parenchymal tissue planes with better precision. In particular, the preoperative 3DR shows the origin and the relationships of the adrenal vein, while with the intraoperative use of ICG, we visualize it through the difference in contrast with the neighboring structures. This aspect is very important because quick identification of the adrenal vein with its subsequent clipping can be made safely, decreasing the operative time. In addition, this experience showed that this technique can be useful during pheochromocytoma surgery because it allows us to early identify the adrenal vein and reduce gland manipulation. A variant of the right adrenal venous anatomy was present in our only case of conversion to the standard treatment group. Two adrenal veins originated from the vena cava, 1 anteriorly and 1 posterior. We think that with the use of ICG and 3DR, we would have handled this situation better. In fact, among the causes of the longer operative time and the greater blood loss in standard treatment is the presence of vascular anatomic variants. To our knowledge, this is the first prospective case-controlled study involving the use of ICG during laparoscopic adrenalectomy and preoperative 3D CT reconstruction.

This study suffers from several limitations: sample size was limited due to the relative infrequency of adrenal masses, a severe restriction of nonurgent surgical procedures, and limited Intensive Care Unit availability during the COVID-19 pandemic (started in March 2020) greatly hampered patients’ enrollment.^49^ ICG may also have limits, such as hepatic fluorescence during right adrenalectomy, hypofluorescence of adrenocortical carcinomas, and very rare cases of anaphylaxis.^11^,^50^ Randomized trials need to be carried out for evaluating the role of these methods in LA. Finally, given the difficulty of this surgery, it would be interesting to evaluate its possible use in the training of young surgeons.

## CONCLUSIONS

According to our results, we can state that the use of intraoperative ICG in LA and preoperative planning with 3DR images is safe and can improve surgical outcomes with a reduction in operative time and blood loss. Finally, this approach is helpful in guiding size and localization analysis and better identification of anatomy (resection margins, gland vascularity, anatomic variants etc.).
